# Anti-Müllerian hormone level is associated with vitamin D receptor polymorphisms in women with polycystic ovary syndrome

**DOI:** 10.1007/s10815-019-01472-3

**Published:** 2019-05-14

**Authors:** Monika Szafarowska, Edyta Dziech, Beata Kaleta, Monika Kniotek, Artur Rogowski, Agnieszka Segiet - Święcicka, Małgorzata Jerzak

**Affiliations:** 1 Department of Gynecology and Oncological Gynecology, Military Institute of Medicine, 128 Szaserow Str, 04-141 Warsaw, Poland; 2 Department of Clinical Immunology, Transplantation Institute, Medical University of Warsaw, Warsaw, Poland; 3 Collegium Medicum, Cardinal Stefan Wyszynski University, Warsaw, Poland; 4 Department of Obstetrics and Gynecology, Mother and Child Institute, Warsaw, Poland; 5 Chair and Department of Experimental and Clinical Physiology, Laboratory of Centre for Preclinical Research, Medical University of Warsaw, Warsaw, Poland

**Keywords:** AMH, Vitamin D, VDR polymorphism, PCOS

## Abstract

**Objective:**

Our study aimed to investigate the relationship between polymorphisms (Apa1, Bsm1, Fok1, and Cdx2) in the VDR gene as well as AMH and AMHR2 genes and their influence on AMH and 25(OH)D levels in PCOS women.

**Study design:**

Seventy-five patients with PCOS and 23 control women were included. Serum AMH and 25(OH)D levels in patients and controls were measured by enzyme-linked immunosorbent assay (ELISA). Polymorphisms in VDR gene Fok1 C/T (rs2228587), Bsm1 A/G (rs1544410), Apa1 A/C (rs7975232), and Cdx2 A/G (rs11568820) polymorphisms as well as AMH G/T (rs10407022) and AMHR2 A/G (rs2002555) were analyzed using real-time PCR.

**Results:**

Analysis of the VDR Cdx2 polymorphism showed a significantly higher frequency of the homozygous GG (mutant) genotype in the PCOS group as compared with the control group (*p* < 0.05). The analysis revealed a statistically significant correlation between the presence of FokI and ApaI polymorphisms and AMH levels in PCOS women (*p* < 0.05). The presence of mutant genotypes (CT, TT) in the Fok1 and (CA, CC) in the Apa1 polymorphisms were associated with higher AMH level in PCOS women (*p* < 0.05). No statistically significant correlations between AMH and AMHR2 polymorphisms and AMH level were found. Moreover, there was no correlation between AMH and 25(OH)D levels in the PCOS or in the control group.

**Conclusion:**

It seems that the elevated AMH level is associated with VDR Fokl and Apal polymorphisms, but not with 25(OH)D levels in PCOS women. Further research is needed to determine the role of VDR polymorphism in AMH level in PCOS.

## Introduction

Polycystic ovary syndrome (PCOS) is one of the most common endocrine disorders in women of reproductive age and the most common cause of infertility due to ovulation problems [1]. PCOS was observed to be associated with an increased risk of pregnancy complications as well as neonatal complications (miscarriages, gestational diabetes, gestational hypertension, preeclampsia, and fetal hypertrophy) [2]. Cardiovascular disease, impaired glucose tolerance and type 2 diabetes, obstructive sleep apnea, and endometrial cancer are significantly more common in women with PCOS [3]. The pathogenesis of PCOS remains largely unexplained. According to the literature, abnormal anti-Müllerian hormone (AMH) levels and significant vitamin D deficiency are responsible for a number of different abnormalities observed in PCOS patients [4].

AMH is one of the most reliable markers of ovarian reserve [5]. It regulates the development of early pre-antral and small antral follicles by reducing their sensitivity to follicle-stimulating hormone (FSH). It also has anti-proliferative and pro-apoptotic activity [6, 7]. It has been postulated that AMH is capable of stimulating the activity of hypothalamic gonadotropin-releasing hormone (GnRH) secreting neurons by enhancing the secretion of luteinizing hormone (LH); thus, the involvement of AMH in the pathomechanism of PCOS-related disorders can be confirmed [8]. Numerous studies confirmed a two- or even a threefold increase in AMH levels in PCOS patients as compared with healthy individuals. Cimino and coworkers suggested that high AMH levels are correlated with the severity of PCOS [9]. However, the etiology of PCOS and the mechanism responsible for elevated AMH levels in PCOS patients remain unexplained, and the maintenance of normal AMH levels appears to play the key role in the management.

After the worldwide epidemic of vitamin D deficiency had been declared [10], numerous studies were conducted to show that vitamin D plays an important role in the metabolic pathways in PCOS women [11]. Vitamin D deficiency was shown to be much more common in PCOS women and was related to the severity of the syndrome [12]. Besides regulation of calcium and phosphate metabolism, the range of vitamin D actions includes pro-differentiation, anti-proliferation, pro-apoptosis, immunosuppression, and anti-inflammation [13]. Vitamin D is proposed to be an important steroid hormone involved in reproduction. Low vitamin D level is correlated with impaired ovarian function and with the increased risk of leiomyomas [14]. In addition, the important role of this hormone in reproduction medicine is confirmed by higher pregnancy rates in women with higher levels of vitamin D [15].

The maintenance of biological equilibrium with regard to proper AMH and vitamin D levels is a very complicated process. In a vast majority of studies, a significant correlation was observed between AMH and vitamin D levels. Naderi and coworkers suggested that vitamin D supplementation leads to increased serum AMH levels [16]. Slightly different results were obtained by Irani and coworkers who showed that administration of vitamin D in women with PCOS may reduce the AMH levels [17]. However, no correlation between AMH and vitamin D levels was observed in some studies [18]. Because of the controversial findings, the role of AMH in pregnancy outcome is uncertain.

The impact of vitamin D on AMH synthesis remains unclear. The active form of vitamin D (calcitriol, 1,25(OH)D) mediates its biological effects by binding to vitamin D receptor (VDR), which acts as a transcription factor. Currently available data showed that *AMH* is a target gene for calcitriol. Krishnan and coworkers identified vitamin D responsive element (VDRE) in the human *AMH* promoter and found that AMH mRNA expression is upregulated in response to calcitriol [19]. Because vitamin D activity is mediated by VDR, analysis of the *VDR* genetic variation may elucidate the role of vitamin D in PCOS. It has been postulated that VDR polymorphisms (*ApaI*, *BsmI*, and *FokI*) may contribute to PCOS susceptibility [20]. The *VDR* gene lies on the long arm of chromosome 12 (12q12-14) and has approximately 200 single-nucleotide polymorphisms (SNPs) [21]. Each polymorphism is named according to the restriction site that was initially used to identify it. The most common allelic variants studied include *VDR* Fok1 C/T (rs2228587), Bsm1 A/G (rs1544410), Apa1 A/C (rs7975232), and Cdx2 A/G (rs11568820) polymorphisms. Polymorphisms in *VDR* can change mRNA stability, efficiency of VDR protein translation, and VDR activity [22]. The presence of FokI polymorphism results in a shortened VDR protein [23, 24]. Moreover ApaI, BsmI, and TaqI may affect gene expression by altering mRNA stability, splice sites for mRNA transcription, or intronic regulatory elements [25]. Presence of different genetic variants in the vitamin D and AMH signaling pathway was also postulated to be a possible cause of reproductive dysfunction in women [26]. Moreover, some data suggest that the *AMH* (rs10407022) and *AMHR2* (rs2002555) polymorphisms result in increased susceptibility to PCOS; however, the outcome is still inconsistent [27].

Considering the significant discrepancy in the available literature data on the effects of vitamin D on AMH levels, we attempted to examine the relationship between the four functionally most relevant *VDR* polymorphisms (Fok1, Bsm1, Apa1, and Cdx2) as well as *AMH* and *AMHR2* gene polymorphisms and their influence on AMH levels in PCOS women.

## Materials

A prospective study was conducted at the Department of Gynecology and Oncological Gynecology of the Military Institute of Medicine, Warsaw, Poland, between 2015 and 2017. The study was approved by the institutional review board. Written informed consent was obtained from all patients.

The study group consisted of 75 women (aged 25–43 years) with polycystic ovary syndrome (PCOS). Meeting two of the following three conditions was required for inclusion:

- Clinical or laboratory features of androgenization;
- Infrequent ovulation or lack of ovulation;
- Polycystic ovaries in ultrasound imaging.

Hirsutism was defined as a Ferriman-Gallwey score of more than 5; the laboratory features were assessed on the basis of free testosterone levels exceeding 3.5 pg/mL. Clinical indicators of infrequent ovulation or lack of ovulation included menstruation disturbances such as irregular menstruation (cycle durations differing by more than 10 days), oligomenorrhea (menstruation occurring with the frequency of 40 days to 6 months), and secondary amenorrhea (no menstruation over a 1-year period).

Ovarian morphology was assessed by means of transvaginal ultrasound. Polycystic ovaries were defined as ovaries with the ultrasound appearance of more than 12 subcapsular follicles (with diameters of 2–9 mm) or as ovaries with total volumes larger than 10 cm^3^ according to the Rotterdam criteria published in 2003 [28].

The control group consisted of 23 subjects (aged 27–42 years) recruited from young, healthy women without disorders in their obstetric-gynecological and internal medical history. None reported any problems with conception; all subjects declared a normal course of pregnancy and delivery. In addition, none of the control subjects was under any medical treatment. Transvaginal ultrasound scans were performed in all patients between day 3 and day 5 of the menstrual cycle to reveal normal morphology of the uterus, endometrium, and appendages. Women on oral hormonal contraception or women with hormonal intrauterine devices were excluded from the study.

## Methods

According to the study protocol, blood samples were collected from patients and controls. Patient demographics and physical examination data were obtained from the medical documentation of patients and patient surveys carried out for that purpose.

### Evaluation of AMH and 25(OH)D serum levels by enzyme-linked immunosorbent assay

Serum AMH and 25(OH)D levels in patients and controls were measured by enzyme-linked immunosorbent assay (ELISA) according to the manufacturer’s instructions (DRG® AMH ELISA, EIA-5738 and 25-OH vitamin D (total), EIA-5396) in duplicate. Chromate 4300 Microplate Reader was used for reading the plates at 450 nm. The results were expressed in ng/mL. According to the reference range of the kit, serum AMH levels of more than 6 ng/mL indicated PCOS; serum 25(OH)D levels < 20 ng/mL were considered deficient.

### DNA isolation and genotyping

Peripheral blood samples were collected in EDTA-coated tubes. Genomic DNA was isolated according to standard procedures in the Blood Mini Kit (A&A Biotechnology) and then stored at − 20 °C. Genotypes of *VDR* Fok1 (rs2228587), Bsm1 (rs1544410), Apa1 (rs7975232), Cdx2 (rs11568820), and AMH (rs10407022) and AMHR2 (rs2002555) were analyzed using a real-time PCR method. Genotyping was carried out with the LightSNiP assay (TIB-MolBiol, Berlin, Germany) by analyzing the melting curves with the LightCycler® 480 system available from Roche Diagnostics. Probes used in the study are commercially synthesized after entering the rs (reference SNP ID) number. Real-time PCR reactions were performed in 96-well PCR plates with cycling conditions as optimized by TIB-MolBiol.

## Statistical analysis

The data collected in this study was summarized using descriptive statistics. In descriptive statistics for categorical variables, counts and percentages were provided. For continuous variables, the distribution was first evaluated using the Shapiro-Wilk test. For normally distributed variables, the mean and standard deviation were reported; otherwise, the median and the 25th and 75th percentiles were reported. Categorical variables were compared using the Fisher test or the chi-squared test depending on category sizes. Normally distributed variables were compared with the Student *t* test, and the Mann-Whitney test was used otherwise. The continuous variables were assessed with Spearman’s rank correlation; correlation coefficient and *p* value were reported. Genotype distribution was tested for Hardy-Weinberg equilibrium, and the difference in genotype frequencies was assessed using a chi-squared test. Statistical significance level was set at 5%. Two-sided tests were used.

Statistical analyses were performed using the R 3.1.2 statistical software pack (R Core Team; (2014). R: A language and environment for statistical computing. R Foundation for Statistical Computing, Vienna, Austria. URL http://www.R-project.org/).

## Results

### Characteristics of the study population

Table 1 presents the clinical and demographic data of female subjects included in the study as obtained from the surveys, history interviews, medical documentation, and clinical examinations.

**Table 1:** Descriptive characteristics of the PCOS group

Variable		Total
*n*		75
Age (years)	Mean (SD)	33.9 (4.0)
Weight (kg)	Median [IQR]	59.0 [55.5, 68.0]
Waist circumference (cm)	Median [IQR]	74.5 [70.8, 80.0]
Height (cm)	Mean (SD)	167.1 (5.6)
BMI	Median [IQR]	21.50 [19.95, 23.50]
HR (bpm)	Median [IQR]	75.0 [68.5, 81.0]
SBP (mmHg)	Mean (SD)	121.0 (10.4)
DBP (mmHg)	Median [IQR]	73.0 [70.0, 77.5]
Glucose (mg/dL)	Mean (SD)	87.4 (7.4)
HDL (mg/dL)	Median [IQR]	63.5 [56.6, 75.6]
TG (mg/dL)	Mean (SD)	82.7 (35.1)

### Assessment of AMH and 25(OH)D levels and their correlations with demographic and clinical factors in experimental and control groups

AMH levels within the study group (PCOS) were statistically significantly higher than those in the control group (8.4 ng/mL vs. 4.3 ng/mL, respectively; *p* = 0.001). Moreover, we observed that vitamin D levels were significantly lower in the PCOS group compared with the control group (14.2 ng/mL vs. 19.60 ng/mL respectively; *p* = 0.008).

The analysis of demographic and clinical factors including age, BMI, fasting glucose level, blood pressure, and lipid profiles revealed no statistically significant associations in the study group (PCOS) between these factors and the determined levels of AMH and vitamin D (*p* > 0.05).

To study the pattern of associations between AMH and 25(OH)D levels in the PCOS and control groups, Spearman’s rank correlation was calculated for these two variables. As presented in Table 2, no statistically significant associations were identified.

**Table 2:** Correlation between AMH and vitamin D level in the PCOS group and the control group

Group	Spearman’s correlation coefficient for association between AMH and 25(OH)D	*p* value
PCOS group	0.006	0.957
Control group	0.051	0.818

### Genotype and allele frequency distribution

The genotype distribution for the *VDR*, *AMH*, and *AMHR2* polymorphisms among PCOS patients and controls was in Hardy-Weinberg equilibrium (*p* > 0.05).

Table 3 presents the allele and genotype frequencies observed in the two study groups. The analysis of the *VDR* Cdx2 (rs11568820) polymorphism showed a significantly higher frequency of the homozygous GG (mutant) genotype in the PCOS group as compared with the control group (*p* < 0.05). However, no significant differences were observed in the comparison of other analyzed genotype frequencies between PCOS patients and controls.

**Table 3:** Comparison of observed allele frequencies and genotype distributions in the PCOS and control groups, testing for Hardy-Weinberg equilibrium in the PCOS and control groups (^HWE^*p* value: *p* value for Hardy-Weinberg equilibrium; PCOS vs. control group *p* value: *p* value for comparison of allele and genotype distributions between PCOS and control groups)

Polymorphism (allele/genotype)	Control (*n* (%))	PCOS (*n* (%))	PCOS vs. control group *p* value
*N*	23	75	
AMH rs10407022			
G:T	24 (52.2):22 (47.8)	80 (53.3):70 (46.7)	> 0.999
GG:TG:TT	8 (34.8):8 (34.8):7 (30.4)	22 (29.3):36 (48.0):17 (22.7)	0.525
^HWE^*p* value	0.491	0.969	
AMHR2 rs2002555			
A:G	39 (84.8):7 (15.2)	124 (82.7):26 (17.3)	0.912
AA:AG:GG	17 (73.9):5 (21.7):1 (4.3)	52 (69.3):20 (26.7):3 (4.0)	0.908
^HWE^*p* value	0.850	0.893	
Fok1 rs2228570			
C:T	35 (76.1):11 (23.9)	117 (78.0):33 (22.0)	0.944
CC:CT:TT	13 (56.5):9 (39.1):1 (4.3)	48 (64.0):21 (28.0):6 (8.0)	0.631
^HWE^*p* value	0.956	0.461	
Bsml rs1544410			
A:G	19 (41.3):27 (58.7)	52 (34.7):98 (65.3)	0.520
AA:GA:GG	4 (17.4):11 (47.8):8 (34.8)	8 (10.7):36 (48.0):31 (41.3)	0.633
^HWE^*p* value	0.999	0.914	
Apal rs7975232			
A:C	35 (76.1):11 (23.9)	109 (72.7):41 (27.3)	0.788
AA:CA:CC	13 (56.5):9 (39.1):1 (4.3)	37 (49.3):35 (46.7):3 (4.0)	0.840
^HWE^*p* value	0.956	0.440	
Cdx2 rs11568820			
A:G	14 (30.4):32 (69.6)	30 (20.0):120 (80.0)	0.200
*AA:GA:GG	*1 (4.3):12 (52.2):10 (43.5)*	*7 (9.3):16 (21.3):52 (69.3)*	*0.018*
^HWE^*p* value	0.641	0.096	

*The results in italics are statistically significant

### Analysis of VDR polymorphisms on AMH and 25(OH)D levels in the PCOS and control groups

**Fig. 1:**
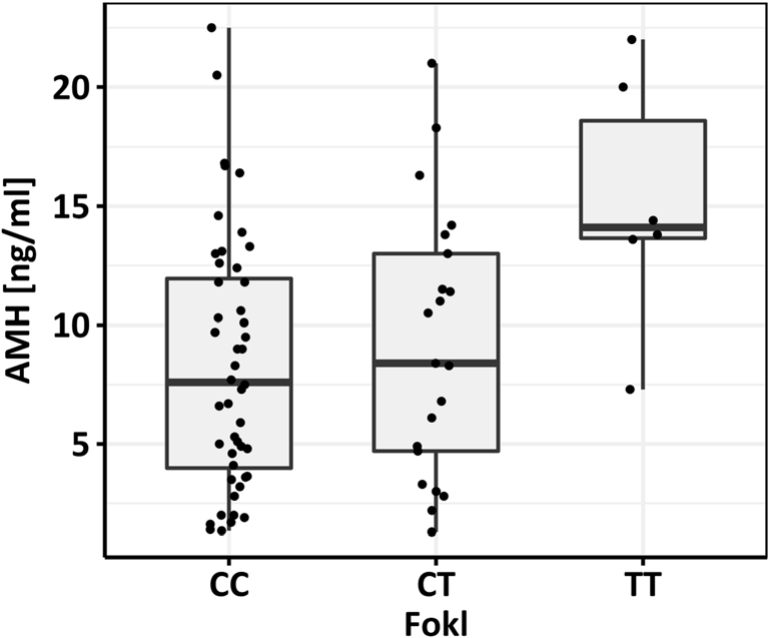
Comparison of AMH levels to Fok1 C/T VDR polymorphism in the PCOS group

**Fig. 2:**
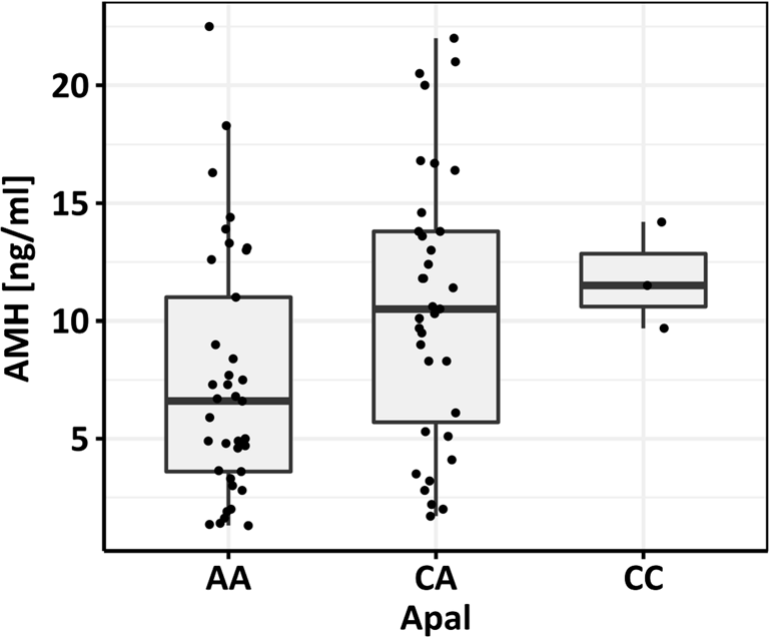
Comparison of AMH levels to Apal A/C VDR polymorphism in the PCOS group

The analysis revealed a statistically significant correlation between the presence of Fok1 (rs2228570) and Apa1 (rs7975232) polymorphisms and AMH levels in PCOS women (*p* < 0.05) (Fig. 1 and Fig. 2). The analysis revealed that the presence of the wild-type (CC and AA, respectively) polymorphic variant was associated with lower AMH values. On the other hand, heterozygous or mutant homozygous genotypes (CT, TT) in the Fok1 and (CA,CC) in the Apa1 polymorphisms were associated with abnormally high AMH levels.

No association of the AMH (rs10407022) and AMHR2 (rs2002555) polymorphisms and the AMH levels was found. Statistical analyses were also performed to study the associations between the abovementioned *VDR*, *AMH*, and *AMHR2* polymorphisms and 25(OH)D levels. We found a statistically significant correlation between the presence of Apa1 (rs7975232) polymorphism and 25(OH)D levels in PCOS women (*p* < 0.05). Presence of the normal (wild-type; AA) polymorphic variant was associated with higher 25(OH)D values. We found no relationship between Fok1, Bsm1, and Cdx2 variants and the 25(OH)D concentrations in PCOS patients and control. Table 4 shows the correlations between the prevalence of individual polymorphisms and AMH and 25(OH)D levels in the PCOS and control groups.

**Table 4:** Comparison of AMH and vitamin D levels to VDR polymorphism in the PCOS and control groups

SNP	AMH (ng/mL) (median [IQR]/mean (SD))		25(OH)D [ng/mL] (median [IQR]/mean (SD))	
	PCOS	Control	PCOS	Control
AMH rs10407022				
GG	8.86 (6.02)	3.75 [1.92, 4.40]	17.00 [13.00, 26.77]	25.38 (14.82)
TG	9.47 (4.98)	3.60 [2.15, 5.97]	13.80 [11.20, 17.62]	19.31 (6.99)
TT	8.70 (6.53)	6.80 [2.90, 8.35]	12.90 [10.20, 19.10]	22.26 (7.53)
*p* value	0.869	0.685	0.112	0.527
AMHR2 rs2002555				
AA	8.00 [4.68, 13.38]	4.40 [2.90, 9.20]	14.65 [11.62, 20.57]	19.20 [16.60, 27.10]
AG	10.00 [3.77, 12.45]	2.00 [1.10, 2.60]	13.50 [11.38, 15.72]	19.60 [15.80, 27.40]
GG	10.10 [8.40, 13.40]	4.90 [4.90, 4.90]	14.30 [12.70, 19.50]	27.40 [27.40, 27.40]
*p* value	0.724	0.260	0.742	0.618
Fok1 rs2228570				
CC	7.60 [3.98, 11.95]	2.90 [0.90, 4.90]	14.10 [11.35, 19.67]	25.10 [17.90, 27.40]
CT	8.40 [4.70, 13.00]	4.90 [4.30, 7.30]	13.70 [11.70, 17.20]	16.60 [13.70, 19.60]
TT	14.10 [13.65, 18.60]	1.10 [1.10, 1.10]	17.00 [15.23, 18.62]	19.60 [19.60, 19.60]
*p* value	*0.029*	0.191	0.753	0.618
Apa1 rs7975232				
AA	6.60 [3.60, 11.00]	3.70 [2.00, 4.90]	16.60 [12.90, 28.20]	19.20 [16.60, 27.10]
CA	10.50 [5.70, 13.80]	4.40 [3.80, 9.20]	11.90 [11.10, 15.75]	19.60 [15.80, 27.40]
CC	11.50 [10.60, 12.85]	0.80 [0.80, 0.80]	11.90 [11.85, 12.25]	11.30 [11.30, 11.30]
*p* value	*0.028*	0.254	*0.013*	0.317
Cdx2 rs11568820				
A	4.80 [3.45, 6.60]	2.60 [2.60, 2.60]	14.90 [12.25, 24.35]	25.10 [25.10, 25.10]
A	10.20 [6.75, 13.85]	6.10 [3.20, 9.73]	11.75 [10.95, 17.25]	19.40 [17.38, 27.02]
GG	9.25 [4.36, 13.38]	3.35 [1.33, 4.40]	4.40 [11.78, 19.67]	18.10 [13.25, 27.40]
*p* value	0.076	0.220	0.543	0.890
Bsm1 rs1544410				
AA	7.50 [3.08, 12.78]	3.30 [2.82, 5.45]	12.20 [11.07, 16.52]	19.30 [13.10, 25.58]
GA	8.70 [4.88, 13.95]	4.40 [4.05, 9.30]	14.10 [11.20, 18.90]	19.60 [18.45, 30.00]
GG	8.30 [4.65, 12.80]	1.85 [1.03, 5.38]	14.80 [11.85, 24.40]	16.65 [12.65, 27.18]
*p* value	0.755	0.288	0.374	0.349

*The results in italics are statistically significant

## Discussion

Vitamin D plays an important role in metabolic pathways associated with PCOS. Its level has been related with the severity of the phenotype of this disorder. However, it should be kept in mind that the prevalence of vitamin D deficiency in the general population of adults is about 20–48% [29]. It has been postulated that vitamin D deficiency among women with PCOS is relatively higher, approximately 67–85% [12]. In accordance with other reports, we confirmed statistically significantly lower vitamin D level in the group of PCOS patients as compared with the control group [18].

One of the most important objectives of this study was to determine the influence of vitamin D and *VDR* gene polymorphisms (Fok1, Bsm1, Apa1, and Cdx2) and *AMH* and *AMHR* polymorphisms on AMH levels in PCOS women. Numerous studies confirmed that the differences in the biological activity of vitamin D are mainly caused by gene expression changes mediated by intracellular vitamin D receptors (VDRs) [30]. VDR, being a member of nuclear receptor family (NR1I1), is believed to be a universal translational complex that controls 3% of the human genome. Following activation with an active form of vitamin D (calcitriol, 1,25(OH)D), VDR forms a heterodimer with retinoid X receptor (RXR) which is enabled to recognize the specific DNA sequences and vitamin D response elements (VDRE) located within the promoter regions of vitamin D–regulated genes [31]. The receptors bind to the regulatory regions of target genes, which is essential for directed changes in transcription. It should be noted that vitamin D receptors were found in many tissues, including ovaries, uterus, and placenta. They play different roles, including regulation of gene networks involved in proliferation and differentiation of cells as well as in the function of the immune system. Thus, the cellular expression of the receptor is very important, and the receptor’s concentration is a key component of cell’s sensitivity to the hormone.

In vitro studies demonstrated that the *AMH* gene is upregulated by vitamin D via functional vitamin D response elements that bind the vitamin D receptor (VDR) [6]. There are multiple reports regarding the relationship between AMH and vitamin D and the impact of vitamin D supplementation on AMH levels [32, 13, 16]. When analyzing the available literature, it is clear that most of the studies revealed that supplementation of vitamin D to women with low AMH levels leads to an increase in these levels, thus improving reproductive outcomes [33]. AMH levels in PCOS women are 2–3 times higher than those in the control group, as was also confirmed in our study (*p* < 0.05) [34]. However, it was demonstrated that too low (< 0.2 ng/mL) as well as too high (> 6.1 ng/mL) levels of AMH, particularly in PCOS women, are associated with poorer reproductive outcomes [35]. Therefore, it is very important to keep the AMH levels within normal range. Several strategies have been proposed to achieve this important goal.

Current data present an inconsistent correlation between vitamin D supplementation and AMH levels. Jukic and coworkers found some evidence that low vitamin D levels were associated with low AMH levels and other biomarkers of ovarian reserve [36]. The association between vitamin D and AMH levels was also confirmed by Dennis and coworkers, who demonstrated a significant seasonal correlation of vitamin D and AMH concentrations [33]. According to the study, higher vitamin D levels observed in the summer season were associated with higher AMH concentrations. In addition, supplementation with vitamin D was found not only to sufficiently replenish seasonal drops in AMH and vitamin D levels but also to increase the overall concentrations of both substances. Similarly, Merhi and coworkers observed a positive correlation between serum concentrations of vitamin D and AMH, thus suggesting a relationship between low ovarian reserve and vitamin D deficiency. However, it should be noted that no such correlation was observed in younger women [13]. In contrast to previous studies, some reports suggest an inverse correlation between vitamin D supplementation and AMH levels. Irani and coworkers demonstrated that administration of vitamin D in PCOS women could decrease AMH levels [17]. The clinical effect was notably positive as it could result from improved folliculogenesis and thus improve reproductive outcomes.

Our study demonstrated no significant correlation between AMH and vitamin D levels. Similarly, Drakopoulos and coworkers aimed at finding potential correlations between serum vitamin D levels and ovarian reserve in infertile women. However, researchers did not find any correlations between AMH levels and antral follicle counts (AFC) in patients with normal vitamin D levels and in patients with vitamin D deficiency [37]. Also, Cappy and coworkers were unable to demonstrate any significant correlation between vitamin D supplementation and AMH levels in women with PCOS diagnosed with vitamin D deficiency [38]. Additionally, Pearce and coworkers, in their study conducted on 340 PCOS women, did not observe any changes in AMH levels secondary to the changes to vitamin D levels [39].

Such a large discrepancy in the available literature data may suggest that the mechanism of action is different and probably depends on *VDR* polymorphisms rather than on vitamin D levels. Xavier and coworkers in their study demonstrated that Taq1 and Bsm1 polymorphisms were independently associated with PCOS [40]. The clinical role of *VDR* polymorphisms is under consideration. Notably, polymorphisms of the *VDR* gene may affect its biological activity by reducing its activity and transcription levels [41]. *VDR* SNPs within the coding regions in ovarian signaling pathways could play an important role in reproductive outcomes as they are responsible for the synthesis of hormone receptors, metabolic enzymes, or transport molecules. Several studies revealed the statistical significant correlations between *VDR* polymorphisms and clinical and biochemical determinants of PCOS. Dasgupta and coworkers show that the Fok1 polymorphism is associated with infertility, while Cdx2 polymorphism was found to be associated with testosterone levels in PCOS [42].

As mentioned above, *VDR* is present within the *AMH* promoter site. Our study suggests a significant correlation between Fok1 (rs2228570) and Apa1 (rs7975232) polymorphisms of the *VDR* gene and AMH levels in PCOS women. To the best of our knowledge, this is the first study of the relationship between serum AMH and *VDR* SNPs in PCOS women. Our study showed that the wild-type variants of Fokl and Apal gene polymorphisms are associated with lower, normal, AMH levels in PCOS women. On the other hand, heterozygous or mutated genes were associated with much higher, abnormal AMH levels. Based on our findings, we hypothesize that the translational complex formed by the VDR gene with Fok1 and Apa1 SNPs within the *AMH* promoter site leads to the enhancement of AMH synthesis in PCOS women.

The etiology of PCOS and the mechanism of elevated AMH levels in PCOS still remain to be further elucidated. Considering the role of AMH with regard to the regulation of ovarian function, it has been postulated that genetic variants in the signaling pathway can be responsible for the impairment of reproductive function of women. Currently available data show that the *AMH* gene polymorphism is connected with the susceptibility and phenotype of PCOS, which suggests that it is one of the factors devoted to the pathogenesis of PCOS [43]. However, in our study we did not find significant differences in the comparison of *AMH* and *AMHR* polymorphism frequencies between PCOS patients and controls. In addition, Wang and coworkers, based on their meta-analysis, demonstrated that there is no association of the abovementioned genetic variants of *AMH* and *AMHR2* with the development of PCOS [9].

In conclusion, Fok1 (rs2228570) and Apa1 (rs7975232) *VDR* polymorphisms seem to be associated with elevated AMH levels in PCOS women, suggesting their possible role in the PCOS pathophysiology. However, the challenge for the future would be further investigation focusing on a molecular mechanism behind *VDR* SNP.
